# Investigation of a miRNA-Induced Gene Silencing Technique in Petunia Reveals Alterations in miR173 Precursor Processing and the Accumulation of Secondary siRNAs from Endogenous Genes

**DOI:** 10.1371/journal.pone.0144909

**Published:** 2015-12-14

**Authors:** Yao Han, Bin Zhang, Xiaoting Qin, Mingyang Li, Yulong Guo

**Affiliations:** Chongqing Engineering Research Center for Floriculture, Key Laboratory of Horticulture Science for Southern Mountainous Regions, Ministry of Education, College of Horticulture and Landscape Architecture, Southwest University, Chongqing, China; CSIR-National Botanical Research Institute, INDIA

## Abstract

MIGS (miRNA-induced gene silencing) is a straightforward and efficient gene silencing technique in *Arabidopsis*. It works by exploiting miR173 to trigger the production of phasiRNAs (phased small interfering RNAs). MIGS can be used in plant species other than *Arabidopsis* by co-expression of miR173 and target gene fragments fused to an upstream miR173 target site. However, the efficiency and technical mechanisms have not been thoroughly investigated in other plants. In this work, two vectors, pMIGS-chs and pMIGS-pds, were constructed and transformed into petunia plants. The transgenic plants showed *CHS* (*chalcone synthase*) and *PDS* (*phytoene desaturase*) gene-silencing phenotypes respectively, indicating that MIGS functions in petunia. MIGS-chs plants were used to investigate the mechanisms of this technique in petunia. Results of 5′- RACE showed that the miR173 target site was cleaved at the expected position and that endogenous *CHS* genes were cut at multiple positions. Small RNA deep sequencing analysis showed that the processing of *Arabidopsis* miR173 precursors in MIGS-chs transgenic petunia plants did not occur in exactly the same way as in *Arabidopsis*, suggesting differences in the machinery of miRNA processing between plant species. Small RNAs in-phase with the miR173 cleavage register were produced immediately downstream from the cleavage site and out-of-phase small RNAs were accumulated at relatively high levels from processing cycle 5 onwards. Secondary siRNAs were generated from multiple sites of endogenous *CHS-A* and *CHS-J* genes, indicating that miR173 cleavage induced siRNAs have the same ability to initiate siRNA transitivity as the siRNAs functioning in co-suppression and hpRNA silencing. On account of the simplicity of vector construction and the transitive amplification of signals from endogenous transcripts, MIGS is a good alternative gene silencing method for plants, especially for silencing a cluster of homologous genes with redundant functions.

## Introduction

Methods to disrupt gene function are very important tools in basic plant science research and in crop improvement. The approaches that have been used for gene knockout or knockdown in plants comprise mainly chemical mutagenesis, physical mutagenesis, insertional mutagenesis, TILLING (Targeting Induced Local Lesions in Genomes), methods based on small RNAs [1], and the recently developed CRISPR (Clustered Regularly Interspaced Short Palindromic Repeats)/Cas (CRISPR-associated) systems [2]. Each approach has both advantages and drawbacks. Gene silencing methods based on small RNAs are low-cost, and have been used for various different purposes and in a variety of plant species. They have been amongst the most popular techniques for the disruption of gene activity in plants [1,3].

The term ‘small RNAs’ usually refers to non-coding RNAs, 20–24 nucleotide (nt) in length, that repress gene expression and function across a range of aspects of plant growth and development. Plant small RNAs comprise mainly microRNAs (miRNAs) and small interfering RNAs (siRNAs). The former originate from precisely processed endogenous hairpin transcripts, leading to the preferential accumulation of one or a few functional small RNAs, whereas siRNAs originate from double-stranded RNAs, yielding a variety of small RNAs that are not uniform in sequence [4]. The first step in small RNA production is performed by Dicer-like (DCL) endonucleases and produces short duplexes with 2-nt 3′ overhangs. Subsequently, one strand from each duplex is loaded onto an ARGONAUTE (AGO) protein, and together with other protein factors they form the so-called RNA-induced silencing complex (RISC). The AGO-bound small RNAs guide the RISCs to target sequences by complementary pairing and regulate target gene activities by transcriptional silencing, cleavage of target transcripts or translational inhibition [5]. In a few cases, the miRNA-mediated cleavage of target transcripts results in the production of a cluster of 21-nt phased secondary small interfering RNAs (phasiRNAs) that are in phase with the cleavage site [6–10]. In other cases, siRNA–target interaction can induce the generation of various secondary siRNA species via transitivity [11–15].

Co-suppression was one of the first observed gene silencing phenomena mediated by small RNAs. In 1990, when the *chalcone synthase-A* (*CHS-A*) gene was overexpressed under the control of the cauliflower mosaic virus 35S promoter in an attempt to intensify the purple color of petunia flowers, variegated or even completely white flowers were observed unexpectedly in many plants [16]. In these transgenic plants, both the expression of the exogenous *CHS-A* transgene and that of the endogenous *CHS-A* gene were suppressed, and the term “co-suppression” was therefore coined. Subsequent studies have proved that the effector molecules in co-suppression are small RNAs [15,17–19]. Co-suppression has been observed in many plant species and it has been exploited in the analysis of plant gene function. However, the gene silencing that is induced by co-suppression is less effective than that induced by the hairpin RNA (hpRNA) and virus-induced gene silencing (VIGS) techniques [20–22].

In hpRNA-induced gene silencing, the hpRNA transgene construct is made by inserting inversely repeated fragments of the target gene between a plant promoter and terminator. A spacer consisting of an intron sequence is often placed between the inverted repeats to stabilize the transgene construct and to enhance the silencing efficacy. This kind of construct is referred to as an intron-hpRNA construct. When RNA is transcribed from hpRNA constructs, a hairpin RNA structure will be formed due to the hybridization of the inverted repeats. The double-stranded region of the hairpin RNA is then processed to produce siRNAs, which guide RISCs to repress the expression of target genes [22]. The hpRNA platform has been widely exploited in plant gene function analysis. However, the construction of an hpRNA transgene vector using conventional cloning methods requires at least two cloning steps and it is time-consuming and laborious [23]. Although novel methods have been described [23–25], the production of an hpRNA vector remains complicated. It is also difficult to stack multiple transgene cassettes [3].

VIGS technology was developed on the basis that the replication of plant viruses can induce the production of siRNAs from viral genomes. Thus, when a viral genome is modified to include a target sequence, siRNAs targeting the host endogenous gene will be produced. For the most common VIGS vector systems in current use, viral cDNAs are modified to facilitate the insertion of host-derived target sequences. The modified viral cDNAs carrying foreign sequences are placed between a plant promoter and terminator in binary Ti vectors [26,27]. The plant is often transformed with this construct using *Agrobacterium*. The gene silencing symptoms can be observed within a few weeks of inoculation [26,28]. The obvious merits of VIGS in gene function analysis are speed and ease of adaption to high-throughput systems. However, the gene silencing phenotype induced by VIGS is usually not heritable. In addition, its applications are restricted by the host range of the virus used to develop the vector system, and symptoms of virus infection can not be avoided in some cases [29].

The artificial microRNA (amiRNA) transgene vector is constructed by replacing the endogenous miRNA and miRNA* sequences in a miRNA precursor with carefully designed amiRNA and amiRNA* sequences, using overlap PCR. The amiRNA precursor is placed behind a plant promoter in binary Ti vectors. When the amiRNA precursor is transcribed in plants, amiRNAs of the desired sequence will be accumulated at high levels, and the target endogenous transcripts would be knocked down [30]. The amiRNA method is thought to have high specificity and the ability to silence multiple genes simultaneously. However, the construction of an amiRNA transgene construct usually needs multiple steps of PCR [30,31].

Recently, three approaches based on phasiRNA synthesis have been used to silence target genes. Firstly, the MIGS method has been used in *Arabidopsis* by expressing a target gene fragment fused to an upstream miR173 target site (miR173ts), and this technique can be applied to other plant species by transforming plants with a vector that co-expresses miR173 and target gene fragments fused to an upstream miR173ts [32]. When the transgene constructs are introduced into the plant, the miR173 mediated cleavage will trigger the spawning of phasiRNAs from the target gene fragments downstream of the cleavage site. The construction of a MIGS transgene vector can easily be accomplished using only a single PCR step. In *Arabidopsis*, the MIGS approach has been proven to be very effective [32], however, because the target sequence used in MIGS is usually 100–500 nt long, many siRNA species are generated, and this increases the chance of off-targeting. To reduce this chance, a secondary approach–the artificial trans-acting small interfering RNA (atasiRNA) technique–was designed [3,33,34]. In an atasiRNA construct, the sequence fused to an upstream miR173ts is engineered by replacing the native siRNA sequences within the TAS locus (*TAS1a*, *TAS1b*, *TAS1c* and *TAS2*) with siRNA sequences designed according to the rules developed for artificial miRNA[30]. The third phasiRNA-based approach employs a 22-nt artificial microRNA complementary to the target gene to mediate widespread RNA silencing [35]. Because of the amplification ability of the silencing signals and the mobility characteristics of siRNAs, the 22-nt amiRNA-directed silencing was thought to be advantageous over hpRNA- and conventional amiRNA-directed silencing strategies with regard to efficiency and dynamic response [35]. Although phasiRNA-based gene silencing approaches have some advantages over other small RNA-mediated gene silencing tools and have been proven to work well in *Arabidopsis*, they have not been well investigated in other plant species.

Because MIGS is the simplest of the phasiRNA-based gene silencing methods, we have employed it to silence petunia *CHS* and *PDS* genes, in order to investigate the feasibility of using this kind of methods in petunia. The results show that *CHS* and *PDS* were each knocked down effectively in transgenic petunia plants. Deep sequencing results demonstrated that the processing of the miR173 precursor was altered in petunia compared with its processing in *Arabidopsis*, and that abundant secondary siRNAs from endogenous transcripts were accumulated in MIGS-chs transgenic plants.

## Materials and Methods

### Plant materials

Petunia (*Petunia hybrida*) cv. ‘Carpet Purple’ and the inbred lines V26 and Mitchell Diploid (MD) were used in this work. Line V26 was kindly provided by Prof. Manzhu Bao (Huazhong Agricultural University, Wuhan, China). Line MD was a generous gift from Prof. David Lewis (New Zealand Institute for Plant and Food Research Ltd, Palmerston North, New Zealand). ‘Carpet Purple’ was obtained from Known-You Seed (China) Co., Ltd (Xiamen, Fujian, China). Surface-sterilized seeds were sown on half-strength Murashige and Skoog (½ MS) [36] semi-solid medium and grown under long-day conditions (16h light / 8h dark) at 25°C. After two weeks, seedlings were cut within the hypocotyl region and shoots were re-rooted in ½ MS semi-solid medium. Four weeks later, young leaves were collected for *Agrobacterium*-mediated transformation. Transgenic and wild-type plants were grown side-by-side under a 16/8-h photoperiod under greenhouse conditions.

### Vector constructs

Vectors were constructed using conventional molecular cloning methods. To make convenient use of MIGS technology in our laboratory, we first constructed a modified MIGS vector pMIGS-T (S1 Fig), based on the structure of MIGS3.1 [32] and a zero-background TA cloning system (ZeBaTA) [37]. All the elements were amplified from pSAT vectors [38] or from the *Arabidopsis* genome, and assembled into pGreenII0229 [39]. In the new vector, the MIGS3.1 Gateway cassette flanked by attR sites was replaced by a ZeBaTA cassette flanked by *Xcm*I sites, making it available for the insertion of PCR-amplified fragments by TA cloning [37]. To construct the MIGS vector targeting the petunia *CHS-A* gene (X14591), the pMIGS-T was digested with *Xcm*I to allow the generation of a single thymidine residue at both 3′ ends of the vector. The T-vector fragment was separated from the 735bp fragment between the two *Xcm*I sites using agarose gel electrophoresis and recovered using a TIANGEN DNA gel extraction kit (TIANGEN Biotech Co., Ltd., Beijing, China). A 463bp *CHS* fragment was amplified from petunia cDNA and at the same time fused with the miR173 target site sequence using the primers CHS-MF and CHS-MR (Table 1). The PCR products were ligated to the T-vector fragments to produce the construct pMIGS-chs (S2 Fig). To make the MIGS vector targeting the *phytoene desaturase* (*PDS*, AY593974) gene, a 325bp fragment was amplified from petunia cDNA and fused with the miR173 target site using the primers PDS-MF and PDS-MR (Table 1), and ligated to the T-vector fragment to produce the construct pMIGS-pds (S2 Fig). All the binary destination vectors were verified by restriction endonuclease digestion and DNA sequencing. The resultant vectors were introduced into *Agrobacterium tumefaciens* strain GV3101 by electroporation.

**Table 1:** PCR primers used in this study.

Name	Sequence (5′→3′)	Comments
CHS-MF	gtgatttttctctacaagcgaagacatagtggtggttgaagtg	For pMIGS-chs construction
CHS-MR	tatctgggagaagagtttgg	For pMIGS-chs construction
PDS-MF	gtgatttttctctacaagcgaaccagatagggtgacagatga	For pMIGS-pds construction
PDS-MR	gagcggcaaacacgaatg	For pMIGS-pds construction
qCHSA-F	ggcgcgatcattataggttc	qRT-PCR
qCHSA-R	tttgagatcagcccaggaac	qRT-PCR
qCHSJ-F	aaagtttagtggaggcattcc	qRT-PCR
qCHSJ-R	tccatactcactcaagacatg	qRT-PCR
qPDS-F	cgagctgaacgaggatggaagtg	qRT-PCR
qPDS-R	ggtactccgactaacttctccaact	qRT-PCR
qUBQ-F	tggaggatggaaggactttgg	qRT-PCR
qUBQ-R	caggacgacaacaagcaacag	qRT_PCR
CHSA5R-1	gtagttcctaaaccttctttggctgag	5′ RACE (round 1) to map cleavage sites
CHSA5R-2	tgagcaatccagaatagagagttccaa	5′ RACE (round 2) to map cleavage sites
CHSJ5R-1	agagacactatggagcacaacagtt	5′ RACE (round 1) to map cleavage sites
CHSJ5R-2	tagagttccagtcagaaatgcccaat	5′ RACE (round 2) to map cleavage sites
35St-3	tgagcgaaaccctataagaaccctaa	5′ RACE (round1) to map miR173 cleavage sites
35St-4	tgggaactactcacacattattctgg	5′ RACE (round2) to map miR173 cleavage sites
RACE5-1	cgactggagcacgaggacactga	General GeneRacer 5′ Primer (round 1)
RACE5-2	ggacactgacatggactgaaggagta	General GeneRacer 5′ Nested Primer (round 2)

### *Agrobacterium*-mediated transformation

The petunia transformation procedure was based on the methods of Napoli et al [16] and Conner et al [40]. Young leaves from aseptic petunia seedlings were used as the explant sources. Glufosinate-ammonium (Basta) at 4 mg/L was used to select the transformed cells.

### Total RNA extraction and real-time RT-PCR analysis

Total RNA extraction, cDNA synthesis and qRT-PCR analysis were carried ou as described previously [41]. For the amplification of *CHS-A* and *CHS-J*, total RNA was isolated from the petals of opening flowers. Specific primers (qCHSA-F and qCHSA-R, qCHSJ-F and qCHSJ-R, Table 1) were synthesized according to Koseki et al [42]. For the amplification of *PDS*, total RNA was extracted from aseptic seedling leaves. Primers qPDS-F and qPDS-R (Table 1) were used. *UBQ* (SGN-U207515) was used as a reference gene. Specific primers qUBQ-F and qUBQ-R were synthesized according to Mallona et al. [43].

### Cleavage site mapping

Mapping of the cleavage sites of tasiRNA on *CHS-A* and *CHS-J* transcripts was carried out as described previously [41]. The gene specific-primers, CHSA5R-1 and CHS5R-2 for *CHS-A*, and CHSJ5R-1 and CHS5R-2 for *CHS-J*, were used (Table 1). For mapping miR173 cleavage sites, PCR was carried out as described above, with gene specific primers 35St-3 and 35St-4 (Table 1), except that the PCR products were resolved on 0.8% w/v agarose gels and the largest fragments were cloned and sequenced.

### Deep sequencing analysis of small RNAs

The library construction and sequence analysis of petunia small RNAs was performed essentially as described previously [41]. Sequencing was carried out on Illumina Hiseq 2500 (1×51 read length). The nucleotide sequence data have been deposited in the NCBI Sequence Read Archive under the accession number SRR2167461.

## Results

### *CHS* gene silencing using MIGS technology in petunia

**Fig 1:**
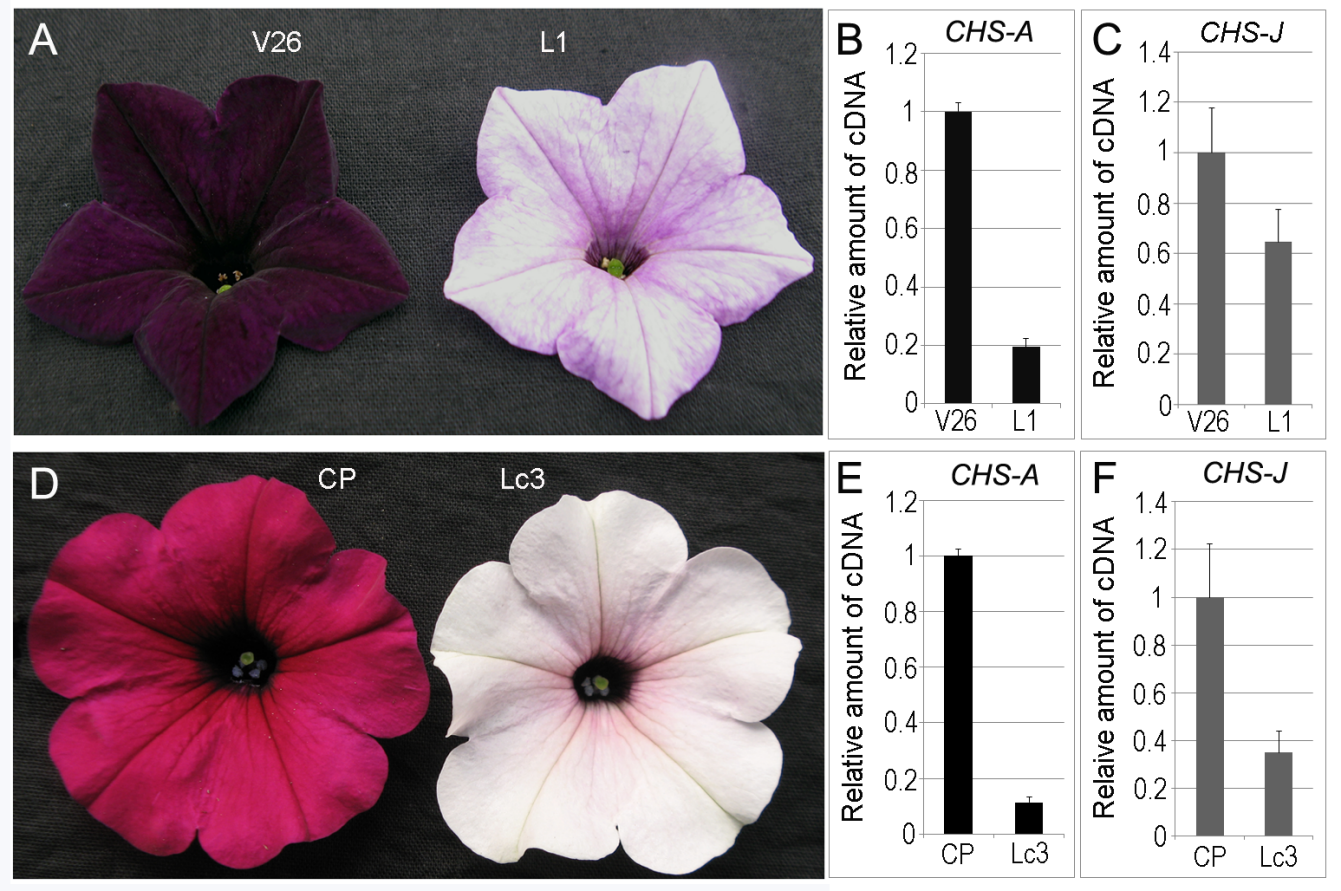
Phenotypes of MIGS-chs transgenic flowers. (A) V26 (wild-type, left) and transgenic (right, L1) flowers. (B and C) qRT-PCR detection of mRNA levels of *CHS-A* and *CHS-J* genes in V26 and in transgenic line 1 (L1). (D) ‘Carpet Purple’ (wild-type, left) and transgenic (right, Lc3) flowers. (E and F) qRT-PCR detection of mRNA levels of *CHS-A* and *CHS-J* genes in ‘Carpet Purple’ (CP) and in transgenic line c3 (Lc3).

In order to investigate the effect of MIGS technology in petunia, we began by suppressing the expression of *CHS* genes. After the pMIGS-chs construct had been introduced into the petunia inbred line V26 and the commercial cultivar ‘Carpet Purple’, transgenic plants showing loss of flower pigmentation were observed in both cases (Fig 1A and 1D). Among the 10 genetic modified lines regenerated from ‘Carpet Purple’, six lines produced flowers with conspicuous color alteration. In the case of V26, nine out of 14 transgenic lines showed alteration of flower color. The flower petals of the MIGS-chs transgenic plants were variegated or nearly white. No “patterned flowers” such as those reported previously in petunia plants cosuppressed for *CHS* [16] were observed in the MIGS-chs transgenic plants in this study.

*CHS-A* (x14591) and *CHS-J* (x14597) are the two main active *CHS* genes in petunia flower tissue. The homology between them is 86% within protein-coding sequences [44]. The accumulation of mRNA in the petals of opening flower was detected using qRT-PCR. For both *CHS-A* and *CHS-J*, mRNA accumulation was reduced in the MIGS-chs transgenic lines (Fig 1B, 1C, 1E and 1F), indicating that *CHS-A* and *CHS-J* mRNAs were degraded.

Because inverted repeats of the transgene in the genome can result in gene silencing, we amplified the genomic DNA from transgenic plants using different single PCR primers to detect inverted repeats of the T-DNA element. No inverted repeat of the T-DNA element was found in the plants subjected to our analysis (data not shown).

### *PDS* gene silencing using MIGS technology in petunia

**Fig 2:**
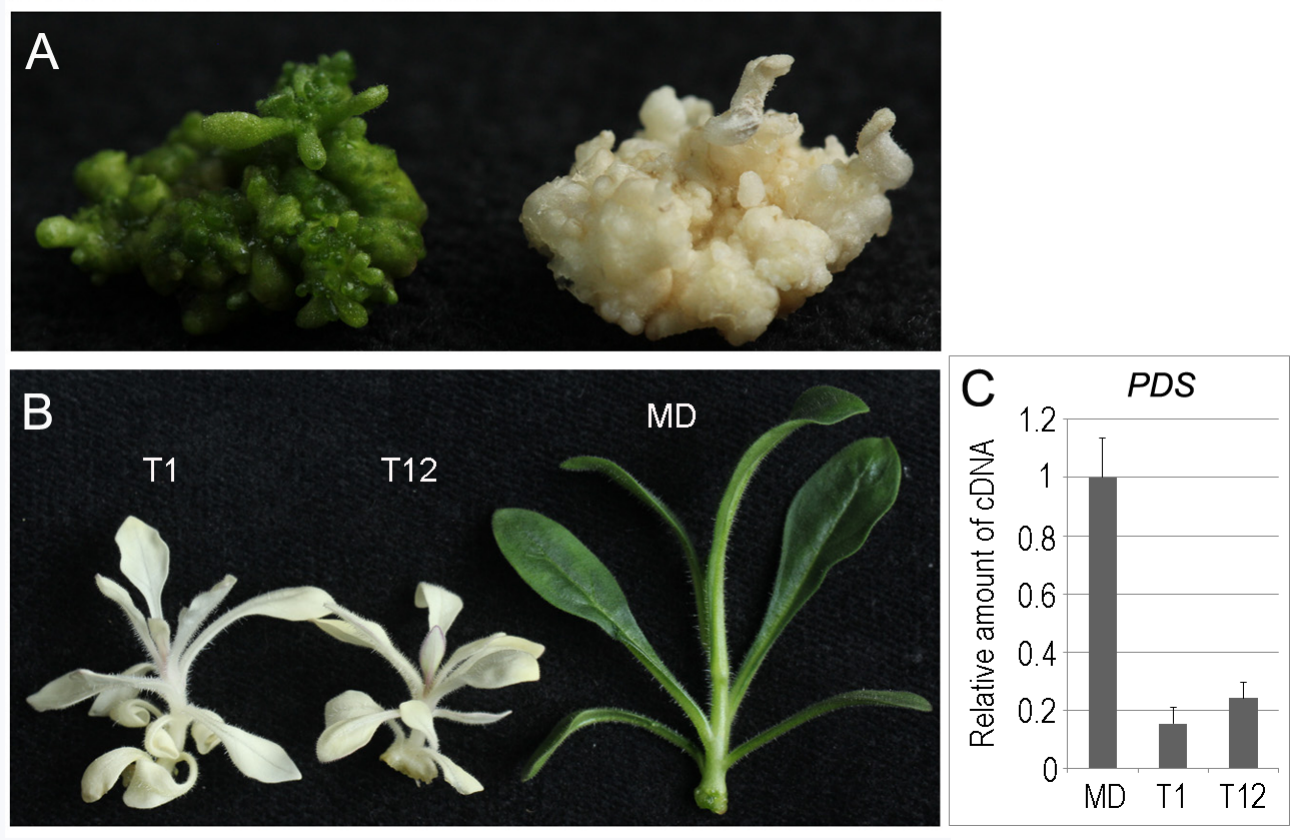
Phenotypes of MIGS-pds transgenic callus and shoots. (A) MD (left) and transgenic (right) petunia callus. (B) MD (right) and transgenic (left) shoots. (C) qRT-PCR detection of mRNA levels of *PDS* genes in MD petunia transgenic lines (T1 and T12).

*PDS* gene is another gene frequently used in silencing research in plants. Because petunia callus is green, we speculated that if the *PDS* gene were silenced, the color of the callus should be altered to white, and the gene silencing effects might be observed earlier than those of *CHS* gene silencing, the effects of which usually manifest as a change in flower color. Prior to this study, however, *PDS* silencing effects in petunia callus had not been reported. After the pMIGS-pds construct had been transferred into MD plants, 90 out of 253 (35.6%) Basta-resistant callus lines showed bleaching (Fig 2A), and many albino shoots were regenerated (Fig 2B). Results from qRT-PCR showed that *PDS* mRNA accumulation had been reduced in the albino transgenic shoots, indicating that the *PDS* gene had been knocked down (Fig 2C).

pMIGS-T (the control vector) was also transferred into MD petunia plants. More than 30 transgenic plant lines were regenerated, and 10 of them were grown to mature plants and allowed to flower and set seed. None of these plants showed morphological and developmental variation compared with the non transgenic plants, indicating that overexpression of *Arabidopsis* miR173 has no obvious detrimental effects on plant development in petunia.

### Detecting miR173-mediated cleavage sites

**Fig 3:**
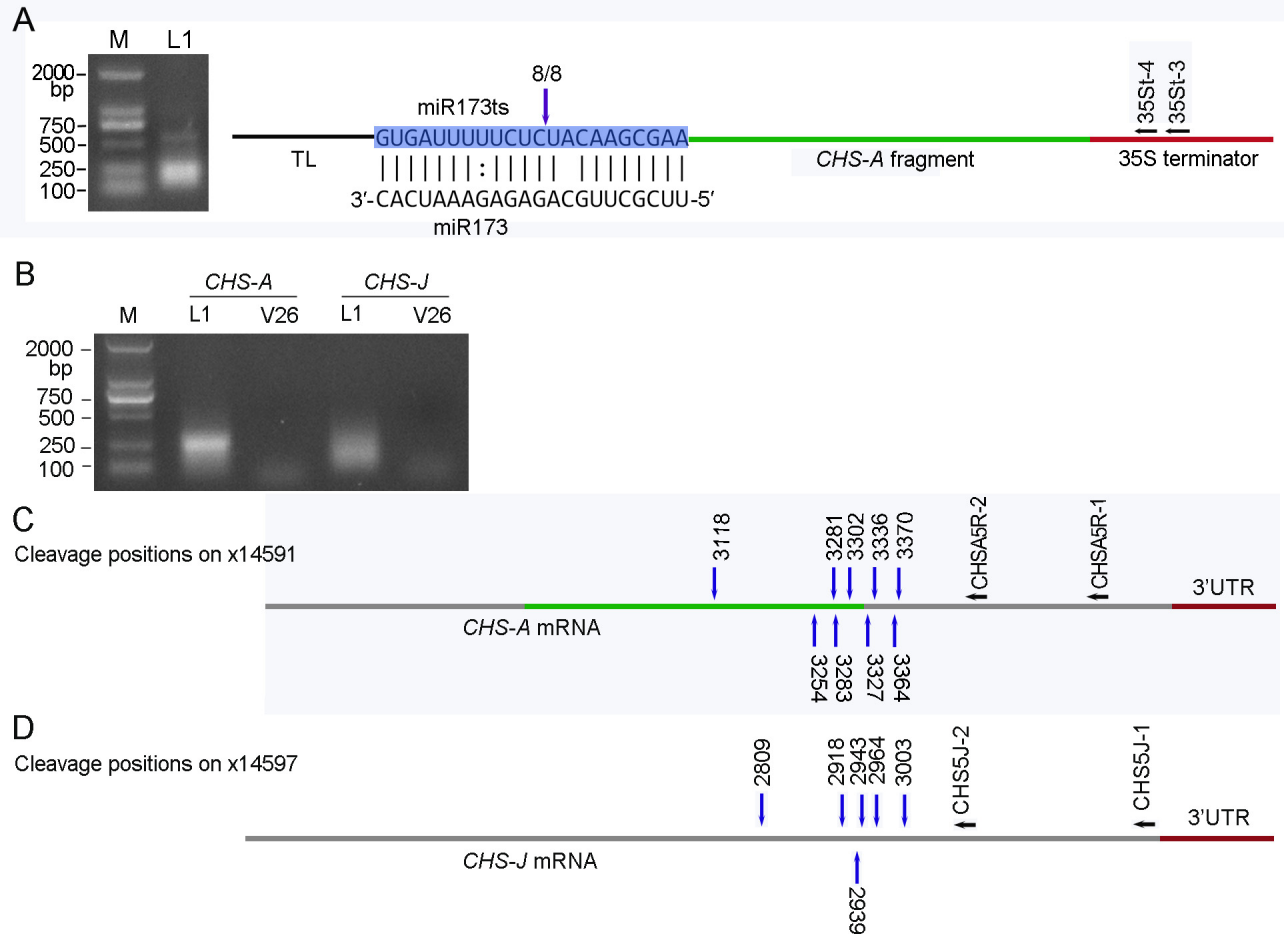
Mapping of miR173- and siRNA-mediated cleavage sites using modified 5′ RLM-RACE. (A) miR173-mediated cleavage of miR173ts_CHS transcripts. The cleavage position and the number of sequenced clones corresponding to the site are indicated by a vertical arrow. (B) Agarose gels showing the products of the mapping of cleavage sites of *CHAS-A* and *CHS-J* mRNAs. (C) Cleavage of *CHS-A* mRNA. (D) Cleavage of *CHS-J* mRNA. The cleavage sites and their corresponding positions on genomic sequences (x14591 and x14597) are indicated by vertical arrows. Horizontal bars represent transcripts. Horizontal arrows indicate the position of gene specific primers used in RACE PCR. TL: tobacco etch virus leader sequence.

To determine whether miR173 guided cleavage of the target transcripts and whether the cleavage occurred at the expected site in MIGS-chs petunia plants, modified 5′ RLM-RACE PCR was carried out using 35S terminator-specific primers. The PCR products presented as an approx. 500bp-long fragment with a smear band (Fig 3A). Sequencing of the largest fragments showed that the miR173ts_CHS transcripts were cleaved at the expected miR173 cleavage site (Fig 3A). This result indicated that the expression of ath-miR173 precursor under a UBQ10 promoter in petunia successfully induced miR173-mediated cleavage, although miR173 was expressed at a relatively low level (see below).

### Detecting cleavages of *CHS* mRNAs

The use of 5′ RLM-RACE PCR to detect cleavages of *CHS-A* mRNAs in pMIGS-chs transgenic plants produced a smear bands (Fig 3B), suggesting that no predominant cleavage sites were generated when the *CHS-A* mRNAs were degraded in petunia plants expressing the pMIGS-chs construct. To further identify the cleavage sites of *CHS-A* mRNAs, PCR products were cloned and sequenced. Random sequencing of nine clones showed that they represented products generated by cleavage of *CHS-A* transcripts at nine different sites (Fig 3C). The results for *CHS-J* were similar to those for *CHS-A* (Fig 3B and 3D). Taken together, these results indicate that the synthesized MIGS construct is effective in petunia.

### MiR173 was expressed at low level in MIGS-chs transgenic petunia plants

**Fig 4:**
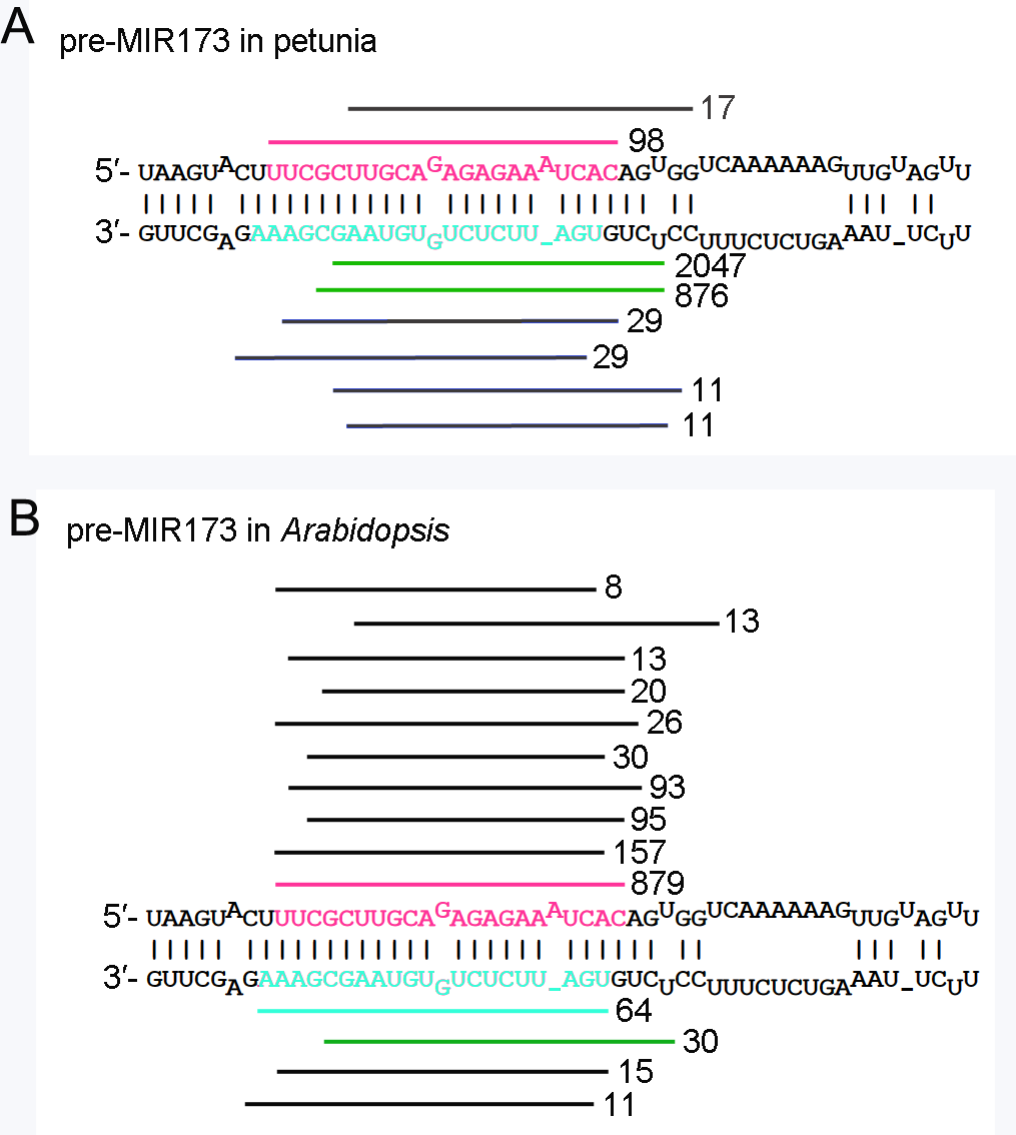
Processing of miR173 precursors. Small RNA sequences from (A) MIGS-chs transgenic petunia petals and (B) *Arabidopsis* were incorporated into the predicted stem-loop of the *Arabidopsis* miR173 precursor. *Arabidopsis* small RNAs were retrieved from the miRBase database (v21). MiR173 is denoted by pink and miR173* by cyan. The read count is indicated beside each small RNA species and only small RNAs cloned more than five times are indicated. Small RNA read counts from *Arabidopsis* totaled 1,485 and from MIGS-chs transgenic petunia the total was 3,190.

Small RNAs in the opening petals of a V26 MIGS-chs transgenic line (L1) were analyzed using the Illumina ‘sequencing by synthesis’ technology. Following quality control, a total of 35,644,137 high quality clean small RNA reads were obtained. The sequences ranged primarily from 18nt to 26nt, with two peaks at 21nt and 24nt (S3 Fig). After collapsing identical reads, 6,131,709 unique small RNA sequences (ranging from 18nt to 32nt) were extracted. Among them, a total of 3,190 reads matched the predicted *Arabidopsis MIR173* stem loop (MI0000217, miRBase). However, only 98 reads (<3 TPM) corresponded to the 22-nt miR173 (ath-miR173-5p) sequence, which was presumed to be the trigger of phasiRNAs production during MIGS, and three reads correspond to the 21-nt miR173* (ath-miR173-3p) sequence. Among the small RNAs that were perfectly matched to the *ath-MIR173* stem loop, the two most frequently occurring small RNAs (designated miR173-3p-1 and miR173-3p-2 in the analysis below) were 20nt and 21nt in length, represented by 2047 and 876 reads, respectively. These two small RNA sequences overlapped with the miR173* sequence and were shifted 4 nucleotides to the 5′ end of the ath-miR173 precursor (Fig 4A). In contrast, analysis of the data retrieved from miRBase (v21, http://www.mirbase.org/) showed that miR173 was the most abundant species among those small RNAs originating from the *MIR173* stem loop in *Arabidopsis* (Fig 4B).

To investigate whether the production of miR173-3p-1 and miR173-3p-2 in petunia was associated with the pMIGS-chs construct, small RNA data for: 1) line V26 expressing artificial microRNA targeted to *CHS* genes (SRP036869), 2) line V26 with co-suppressed *CHS* genes (GSM346607), and 3) wild-type petunia (GSM433598), were searched using the miR173-3p-1 sequence. No small RNA identical to miR173-3p-1 was identified. Secondly, miRBase21 was searched using miR173-3p-1 to determine whether this small RNA might exist in other organism. Except for the ath-miR173 precursor, no sequences showing extensive complementarity to miR173-3p-1 were found. These results indicate that, when introduced into petunia, the ath-miR173 precursor was not processed in exactly the same way as that it is processed in *Arabidopsis*.

### In-phase and out-of-phase small RNAs were spawned from the entire polyadenylated region downstream of the miR173 cleavage site

**Fig 5:**
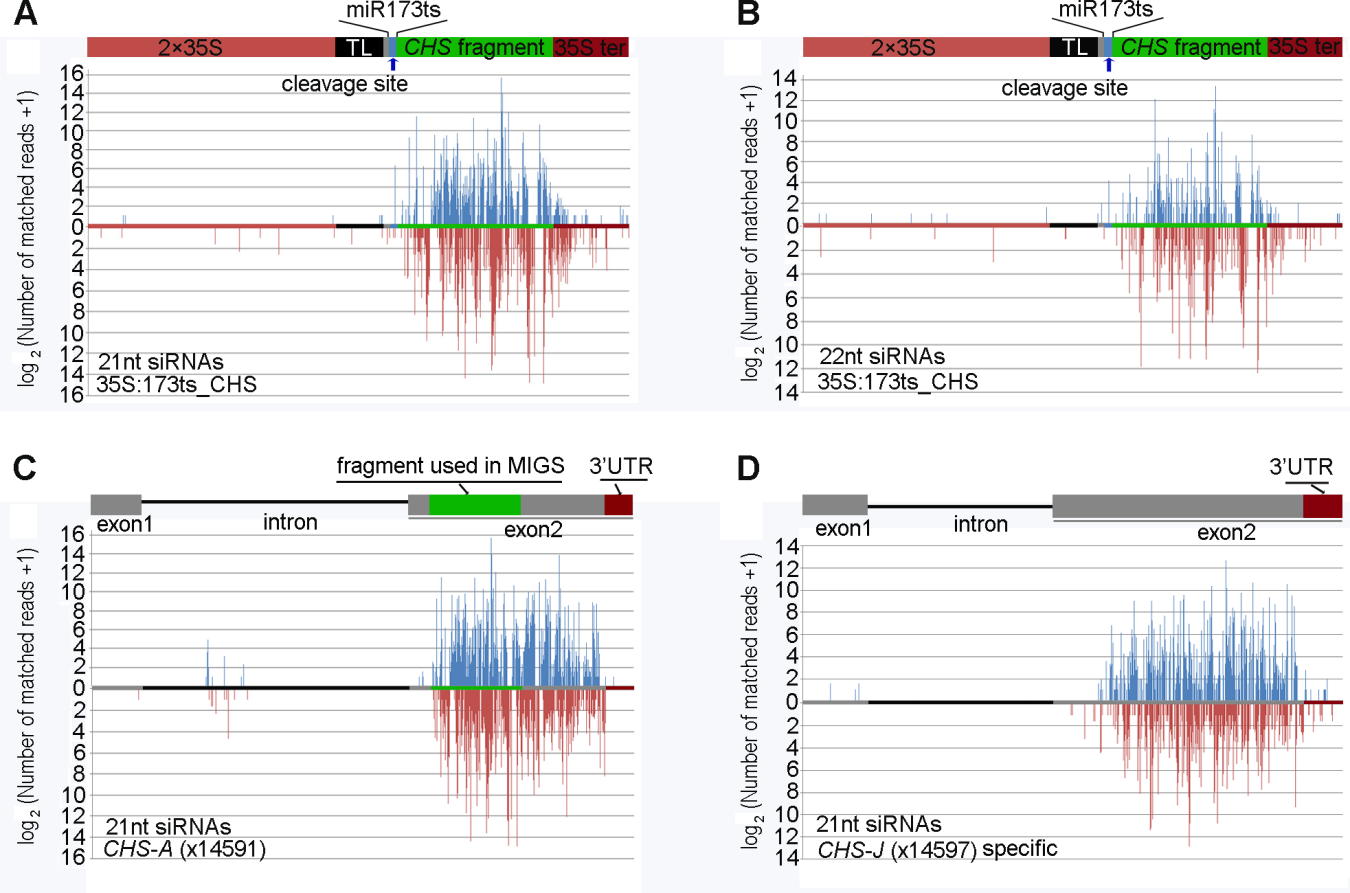
Position and abundance of small RNAs matching the miR173ts_CHS transcripts, endogenous *CHS-A* and *CHS-J*. (A) 21-nt and (B) 22-nt siRNAs mapped onto the miR173ts_CHS transcripts. (C) 21-nt siRNAs mapped onto the *CHS-A*. (D) 21-nt siRNAs specific for *CHS-J*. Vertical bars above and below the horizontal bar (x-axis) denote small RNAs originate from sense and antisense dsRNA strands, respectively.

Mapping 21nt RNAs to the miR173ts_CHS expression cassette (allowing only perfect matches) showed that abundant small RNAs were generated and originated primarily from the region downstream of the miR173 cleavage site (Fig 5A). When small RNA clones with more than 5 read counts were considered, none was found in the TL region (located upstream of the miR173 cleavage site), and only 3 clones (one with 7 reads and two with 5 reads) were mapped to the 35S promoter region. These results indicate a predominantly 5′ to 3′ transitive spread of phasiRNAs initiated as a result of the miR173 cleavage.

**Fig 6:**
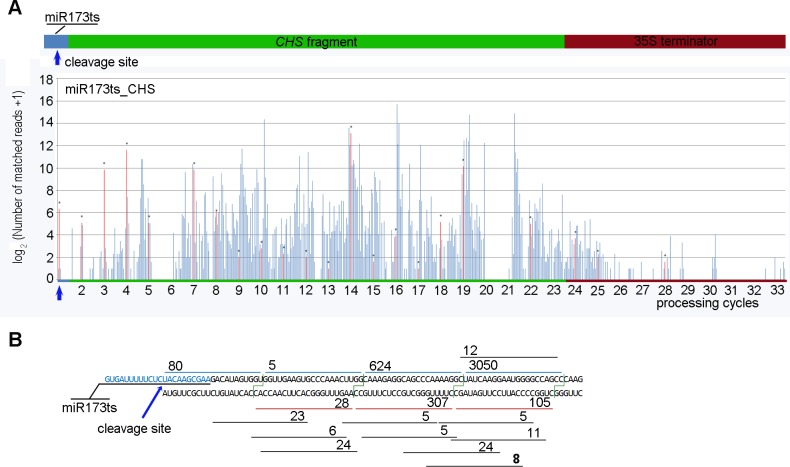
Phase distribution of small RNAs. (A) Phase distribution and abundance of 21-nt small RNAs mapped onto the miR173ts_CHS transcripts. Paired sense and antisense 21-nt RNAs with 2-nt 3′ overhangs are consolidated to one small RNA unit. Processing cycles augment at 21-nt intervals. The first phase position in each processing cycle is in-phase with the miR173 register, and is highlighted in red and indicated by stars if it is populated by at least one small RNA read. (B) Small RNAs mapped onto the first four processing cycles downstream from the miR173 cleavage site. The short horizontal lines indicate 21-nt small RNAs. Their corresponding read counts are shown above each line. Red and blue lines denote those small RNAs in phase with the miR173 register with respect to sense and antisense dsRNA strands, respectively. Only small RNAs cloned more than 5 times are indicated.

Consistent with reports that miR173 cleavage of target transcripts causes the production of phased siRNAs downstream of and in-phase with the miR173 cleavage site[9,10,45], flowers of the MIGS-chs transgenic petunia plants generated phased 21-nt siRNAs immediately downstream from the miR173 cleavage site and extending for five continuous processing cycles (Fig 6A). Between 1 and 25 cycles, 21-nt RNAs in-phase with the miR173 register populated 21 cycle positions. However, only between 1 and 4 processing cycles were the majority of the 21-nt RNA reads in-phase with the cleavage site (Fig 6A and 6B), and from the fifth processing cycle to the end of the 35S terminator region, the 21-nt siRNAs were mostly out-of-phase with the miR173 cleavage site (Fig 6A and S4A Fig). Overall, the in-phase:out-of-phase ratio of 21-nt RNAs produced from the miR173ts_CHS transcripts was about 1:16. The 35S terminator region following the *CHS-A* fragment also generated siRNAs, albeit at much lower levels (Figs 5A and 6A), indicating that the whole of the polyadenylated regions of the miR173ts_CHS transcripts were converted to double-stranded RNAs and used to spawn secondary siRNAs.

Because some reports have suggested that 22-nt RNAs trigger phasiRNA biogenesis in plants [6,46,47], 22-nt RNAs that were perfectly complementary to the miR173ts_CHS expression cassette were searched for and identified. The results showed that, in addition to producing 21-nt RNAs, MIGS-chs transcripts also spawned abundant 22-nt RNA species, with 11049 reads for the most abundant (Fig 5B).

### Transitive small RNAs were produced from the endogenous *CHS-A* and *CHS-J* transcripts

When 21-nt small RNAs were mapped onto the *CHS-A* genomic sequence (allowing perfect matches), it was found that small RNAs were also produced from regions downstream of the fragment used to make the pMIGS-chs construct (Fig 5C), and populated each of the 21 phases (S4B Fig). Small RNAs with read counts of 1–15005 were identified downstream of the target fragment, and only a few species, with 1–29 read counts, were identified upstream of the target fragment, mostly from the intron and promoter regions (Fig 5C and data not shown), indicating a 5′ to 3′ transitive spread.

Small RNAs were also mapped onto petunia *CHS-J* to discover whether abundant small RNAs that were specific to this gene could be identified. Small RNAs that were perfectly complementary to *CHS-J* and that contained at least one mismatched nucleotide to *CHS-A* were regarded as *CHS-J* specific small RNAs. The results showed that abundant *CHS-J* specific small RNAs were produced in the MIGS-chs plants. A total of 836 *CHS-J* specific 21-nt siRNA species were identified, and the most abundant gave 7560 reads (Fig 5D). Small RNAs that mapped onto *CHS-J* populated each of the 21 phases (S4C Fig).

## Discussion

The MIGS technique is a straightforward and highly efficient gene silencing method in *Arabidopsis* [32]. To make use of this tool conveniently in our laboratory, we modified its original vector system to produce pMIGS-T, a vector that allows the insertion of a PCR-amplified target fragment by TA cloning. Because the original MIGS vector system was based on Gateway cloning system, the two-step cloning required is expensive and complicated. To investigate the effects of MIGS technology in petunia, pMIGS-chs and pMIGS-pds constructs were produced to silence *CHS* and *PDS* respectively. When pMIGS-chs was expressed in petunia V26 and ‘Carpet Purple’, the transgenic flowers showed a *CHS*-silencing phenotype, and the accumulation of *CHS-A* and *CHS-J* full-length transcripts was reduced; RLM-RACE PCR results showed that the miR173 target sequences were cleaved at the predicted site, and the *CHS-A* and *CHS-J* transcripts were cut at different sites along their respective transcripts. Phased small RNAs were identified immediately downstream from the miR173 cleavage site. When pMIGS-pds was expressed in petunia MD, the transgenic callus and shoots showed a *PDS*-silencing phenotype; when the control vector (pMIG-T) was expressed in MD plants, no obvious developmental alteration was observed. These results indicate that the MIGS technique based on *Arabidopsis* miR173 induced phasiRNA synthesis works well in petunia, and can be used in petunia gene silencing research.

Unexpectedly, the processing of the *Arabidopsis* miR173 precursor did not spawn miR173 at high frequency in transgenic petunia. In contrast, in MIGS-chs transgenic petunia plants, miR173-3p-1 and miR173-3p-2, which are produced at low frequency in *Arabidopsis*, accumulated respectively at levels more than 20- and 7-fold higher than miR173. Two factors may contribute to the alteration of miR173 precursor processing. First, the original promoter and terminator of *Arabidopsis MIR173* were replaced by a UBQ10 promoter and an OCS terminator in the transgenic petunia plants, which would result in alterations of the 5′ and 3′ regions flanking the miR173 precursor, and these changes may have affected the processing. Because pri-miR173 is a conventional (not a ‘long fold-back’) pri-miRNA, it should be processed from base to loop [48], and the sequence below the miRNA/miRNA* duplex may perform some roles during the generation of miR173. Secondly, subtle differences may exist between species in the machinery of miRNA processing. In support of this explanation, it was observed that when an artificial microRNA precursor based on the *Arabidopsis MIR319a* backbone was expressed in petunia, undesired small RNAs were over-accumulated; some of these had previously been found to be accumulated at low frequency in the products of ath-miR319a precursor processing and some of them were new species [41]. The fact that the processing of both *Arabidopsis* conventional (pre-miR173) and ‘long fold-back’ (pre-miR319a) miRNA precursors was altered in transgenic petunia strongly supports the speculation that there exist subtle differences between different plant species in the machinery of miRNA processing. At present, our knowledge of the processing of microRNA in plants other than *Arabidopsis* is limited. To produce a desired miRNA in a given plant species efficiently, it may be worth recommending the use of its native microRNA precursor as a backbone.

Interestingly, in MIGS-chs petunia plants, although miR173 was not accumulated at high levels as expected, it did initiate phasiRNA production and resulted in obvious *CHS* gene silencing effects. It seems that even a low level of miR173 is sufficient to trigger gene silencing effects in MIGS transgenic plants.

According to the mechanism of the MIGS technique, miR173-mediated cleavage would trigger the production of 21-nt small RNAs in-phase with the miR173 cleavage site [32]. Indeed, small RNAs in-phase with the miR173 register were generated immediately downstream from the miR173 cleavage site and extended for 5 continuous processing cycles (Fig 6A). However, from processing cycle 5 onward, out-of-phase small RNAs accumulated much more than in-phase ones. Out-of-phase small RNAs have been detected at the well-characterized tasiRNA loci in *Arabidopsis*; phase drift occurred after several DCL4 processing cycles of several tasiRNA precursors. For example, Howell et al. [49] found that the phase-forward:initial phase ratio of siRNAs at *TAS1c* was 1:5 between cycles 1 and 5, and 22:1 between cycles 6 and 10. The phase-forward:initial phase ratio of siRNAs at *TAS3a* was 1:3 between cycles 1 and 6, and 32:1 at cycles 7 and 8. For *TAS3c*, the *ARF3*/*ARF4* transcript-interacting tasiR2141-like sequence was detected in-phase with the miR390 cleavage site, but for *TAS3b* it was in the +4 forward phase.

One reason for phase-forward drift could be the inaccuracy of processing by DCLs, which has been found to result in non-21-nt RNAs [49]. In the present study, the ratio of 21-nt to non-21-nt RNAs (20 to 24-nt) was about 4:1 for the miR173ts_CHS expression cassette. It is possible that misprocessing effects from each cycle may be cumulative, resulting a drift of more than one nucleotide as the number of processing cycles increases. A second explanation for out-of-phase siRNA production might be cis-acting siRNAs. A cis-acting siRNA, 3′D10(−), was identified from the *TAS1c* locus. This tasiRNA targets and cuts *TAS1c* transcripts itself, and sets a new register for siRNA production [7]. Thirdly, it is very likely that the miR173-induced siRNAs would cut endogenous target transcripts and trigger the spawning of secondary siRNAs. Because the fragment producing the initial phasiRNAs is a part of the transcript spawning the secondary ones, it is impossible to exclude from the data the secondary siRNAs that have originated from the endogenous genes when we identify the phase position of a small RNA. The secondary siRNAs from endogenous genes would increase the ratio of siRNAs that are out-of-phase with the miR173 register.

The results showed that siRNAs were produced from the *CHS-A* transcripts downstream of the target fragment and abundant *CHS-J*-specific siRNAs were generated in MIGS-chs petunia plants. This clearly demonstrates that miR173-induced “primary” siRNAs can initiate transitivity silencing and induce the production of “secondary” siRNAs from endogenous genes in petunia. It has been reported that tasiRNAs can function *in trans* to induce a tasiRNA cascade, and produce a cluster of secondary siRNAs in-phase with the cleavage site of an initial siRNA [49–51]. In contrast, the siRNAs from the flowers of MIGS-chs plants populated each of the 21 phases in miR173ts_CHS, *CHS-A* and *CHS-J* transcripts (S4 Fig). In *Arabidopsis*, transitivity silencing plays an important role in hairpin transgene-induced silencing and co-suppression. The DCL2 protein, which generates 22-nt siRNA, plays an essential role in the production of secondary siRNAs [11,12]. In MIGS-chs petunia plants, the miR173-induced “primary” siRNAs may act in the same manner as the “primary” siRNAs in co-suppression and hpRNA silencing and to initiate to the spawning of “secondary” siRNAs from endogenous genes in MIGS-chs petunia plants. Abundant 22-nt siRNAs were produced from the miR173ts_CHS transcript (Fig 5D). They may be produced by a DCL2-like protein and may function in transitive silencing.

The production of a variety of siRNA species would simultaneously increase both the efficiency of gene silencing and the chance of off-target effects. However, off-target effects of siRNA do not seem to be an acute problem in plants [52]. Part of the reason may be that the action of siRNAs and miRNAs requires high levels of complementarity in plants and they will therefore tend to target the same specific genes [52]. In addition, plants appear to have mechanisms to prevent the widespread amplification of RNA silencing [12,49]. In the context of gene family silencing, transitive amplification of the silencing signal is an advantage of the MIGS technique. Given that many plants are polyploid or have experienced partial polyploidization at some stage of evolution, gene duplication and functional redundancy is frequently a problem in plant gene function analysis. We can therefore first use MIGS to knock down a cluster of homology genes to investigate their overall probable functions, and then exploit more specific technologies, such as amiRNA and CRIPR/Cas9, to identify more precisely the functions of selected genes.

## Supporting Information

S1 Fig A schematic diagram illustrating miRNA-induced gene silencing (MIGS) of *CHS* genes. (TIF)

S2 Fig Vectors used in this study. (TIF)

S3 Fig Size distribution of small RNA clones from MIGS-chs transgenic petals. (TIF)

S4 Fig Distribution of phased small RNAs. (A) Small RNAs matching the miR173ts_CHS transcripts. (B) Small RNAs matching the endogenous *CHS-A* exon2. (C) Small RNAs matching the *CHS-J* exon2. The locations of the miR173 cleavage site are indicated by vertical blue arrows. Paired sense and antisense 21-nt RNAs with 2-nt 3′ overhangs are consolidated to one small RNA unit. The first nucleotide of “phase 1” corresponds to the first nucleotide of the 3′ miR173 cleavage fragment in (A), and to the first nucleotide of *CHS* exon2 in (B) and (C). Horizontal blue lines represent regions generating three or more tandem 21-nt small RNA units.
(TIF)
