# Supplementary figures and images for: Investigation of a miRNA-Induced Gene Silencing Technique in Petunia Reveals Alterations in miR173 Precursor Processing and the Accumulation of Secondary siRNAs from Endogenous Genes
Source: PLoS One. 2015 Dec 14;10(12):e0144909. doi: 10.1371/journal.pone.0144909 (PMC4701714; doi:10.1371/journal.pone.0144909)

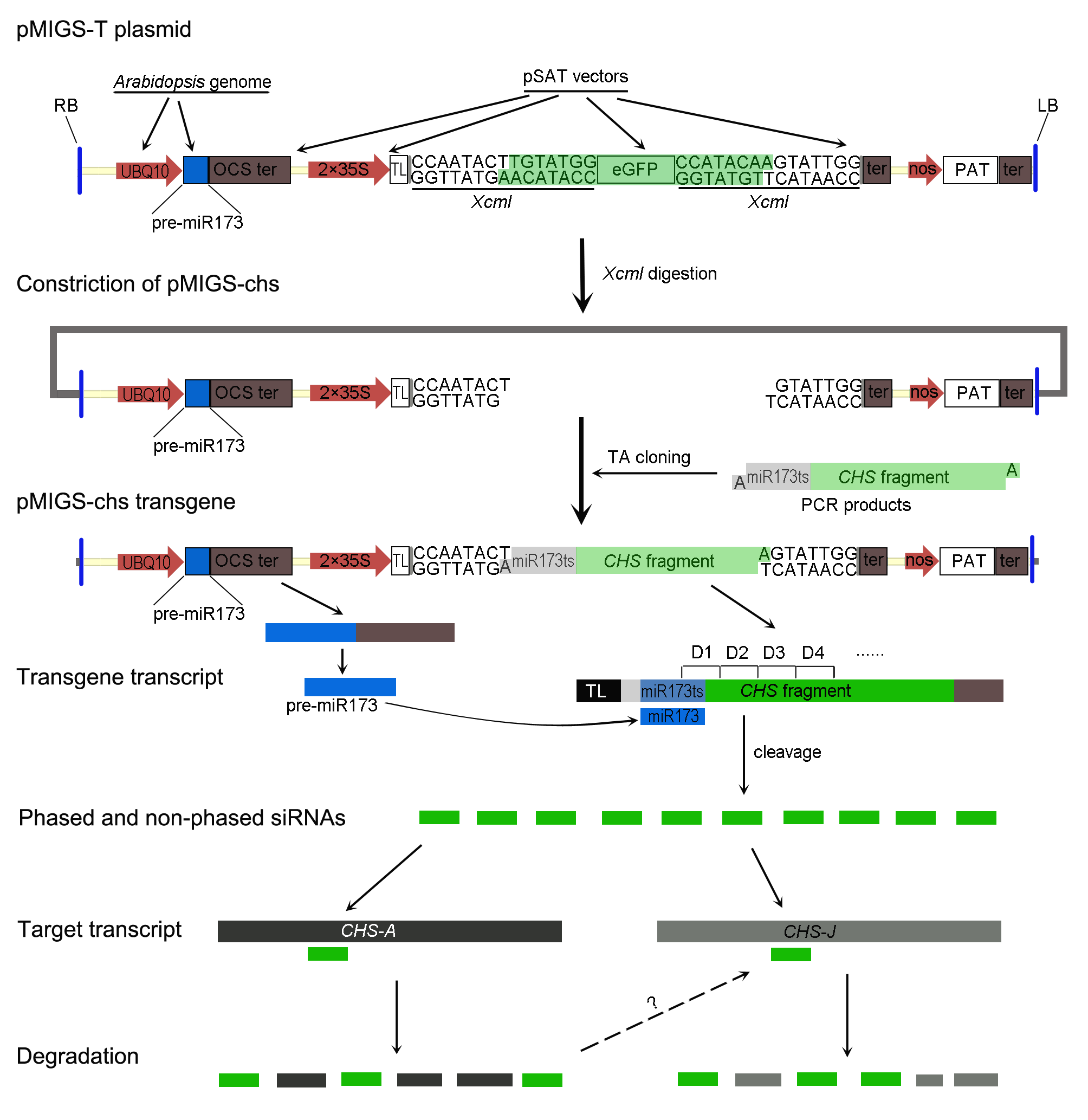

Supplement: S1 Fig — (TIF) [file pone.0144909.s001.tif]

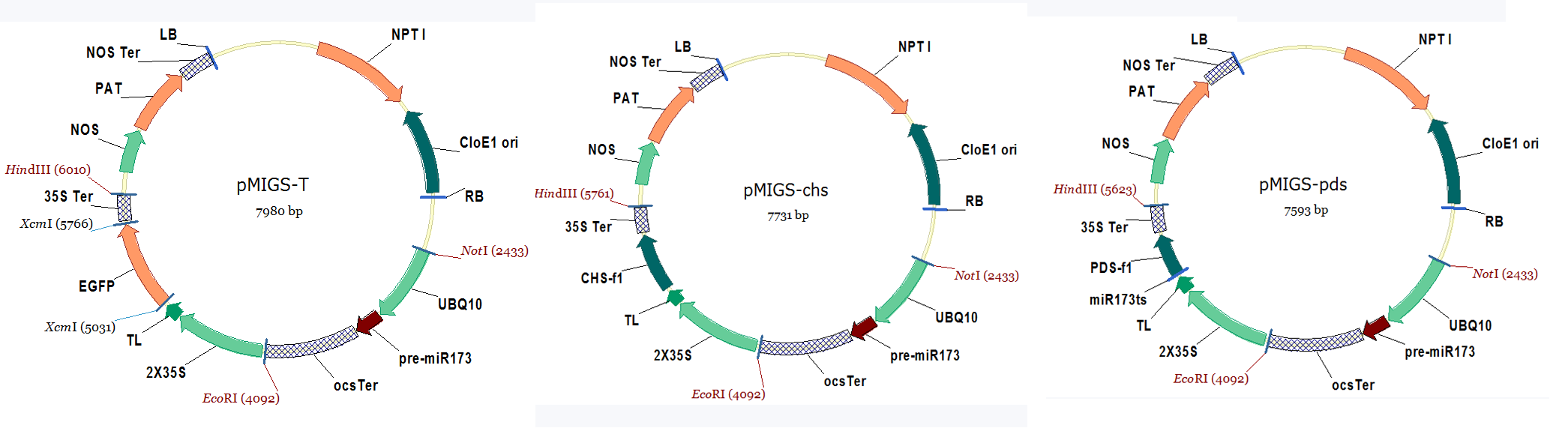

Supplement: S2 Fig — (TIF) [file pone.0144909.s002.tif]

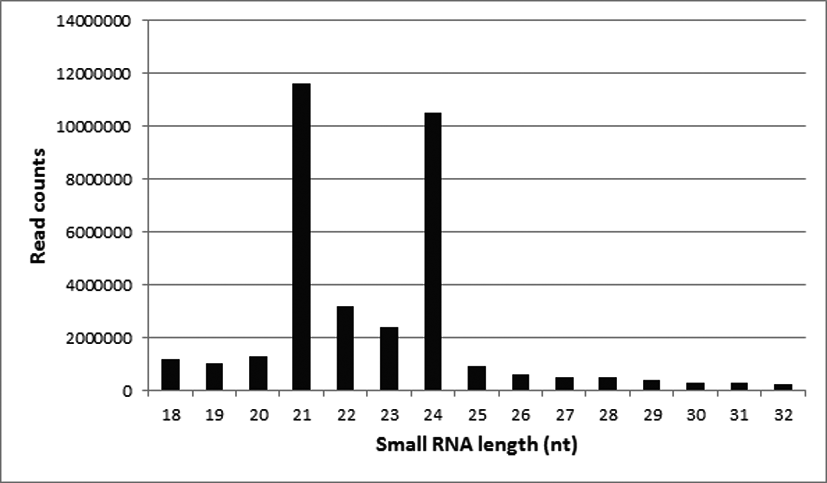

Supplement: S3 Fig — (TIF) [file pone.0144909.s003.tif]

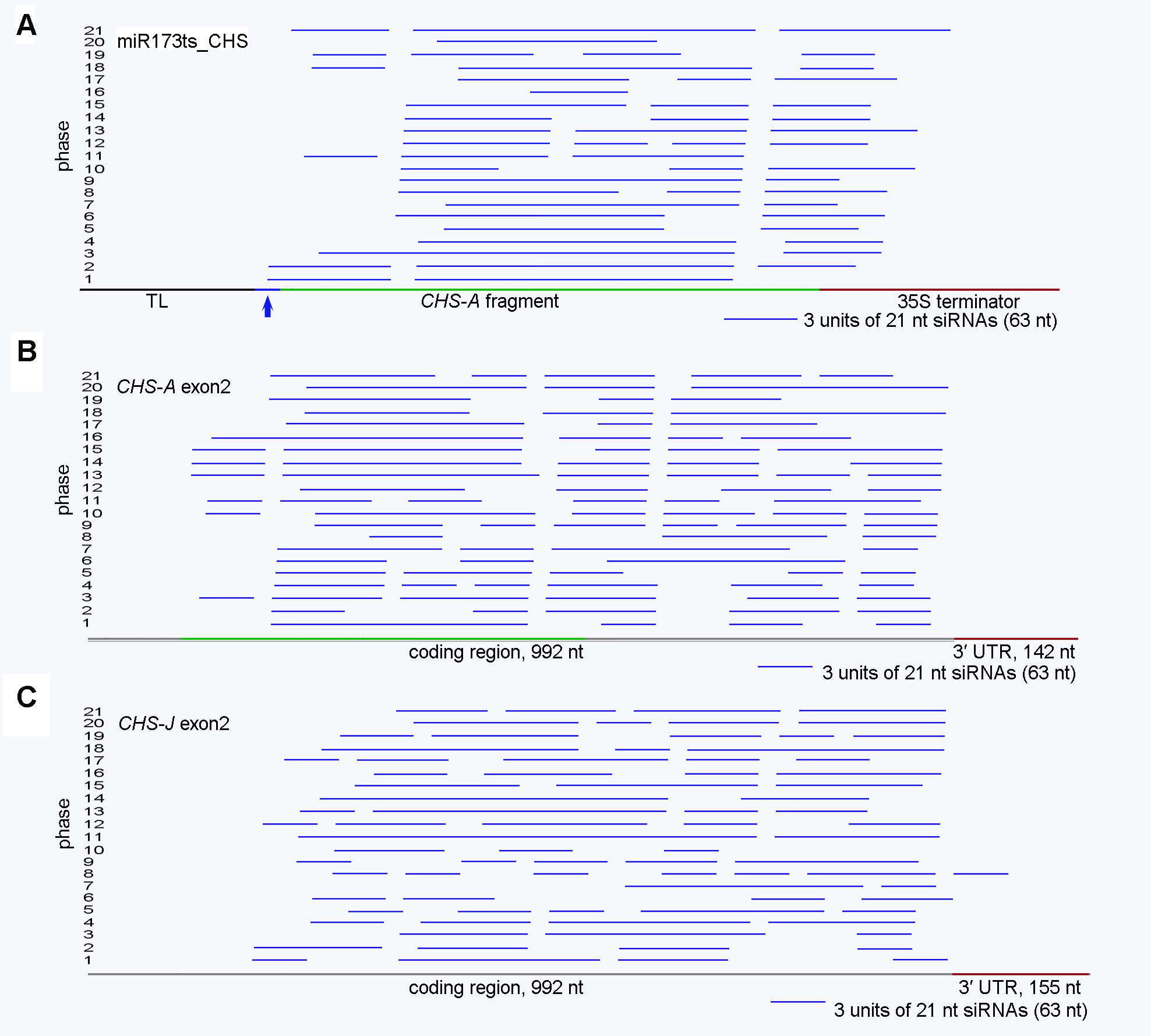

Supplement: S4 Fig — (A) Small RNAs matching the miR173ts_CHS transcripts. (B) Small RNAs matching the endogenous CHS-A exon2. (C) Small RNAs matching the CHS-J exon2. The locations of the miR173 cleavage site are indicated by vertical blue arrows. Paired sense and antisense 21-nt RNAs with 2-nt 3′ overhangs are consolidated to one small RNA unit. The first nucleotide of “phase 1” corresponds to the first nucleotide of the 3′ miR173 cleavage fragment in (A), and to the first nucleotide of CHS exon2 in (B) and (C). Horizontal blue lines represent regions generating three or more tandem 21-nt small RNA units. (TIF) [file pone.0144909.s004.tif]
